# Three years’ interventional neurology experience in Turkey with the Thrombite thrombectomy device in large vessel occlusion in the anterior circulation: safety, efficacy, and clinical outcome

**DOI:** 10.3389/fneur.2024.1320510

**Published:** 2024-05-03

**Authors:** Çetin Kürşad Akpınar, Erdem Gurkas, Atilla Ozcan Ozdemir, Hasan Doğan, Ayşenur Önalan, Serhan Yıldırım, Zülfikar Memiş, Emrah Aytaç, Bilgehan Atılgan Acar, Muhammed Nur Öğün, Özlem Aykaç, Zehra Uysal Kocabaş, Türkan Acar, Halil Alper Eryılmaz, Berkhan Topaktaş

**Affiliations:** ^1^Samsun Tranining and Research Hospital, Stroke Center, Department of Neurology, Samsun University, Samsun, Türkiye; ^2^Kartal Dr. Lütfi Kırdar City Hospital, Stroke Center, Department of Neurology, Health Sciences University, İstanbul, Türkiye; ^3^Eskisehir Osmangazi University Hospital, Stroke Center, Department of Neurology, Eskişehir Osmangazi University, Eskişehir, Türkiye; ^4^Kocaeli City Hospital, Stroke Center, Kocaeli, Türkiye; ^5^Haseki Training and Research Hospital, Stroke Center, Department of Neurology, Health Sciences University, İstanbul, Türkiye; ^6^Fırat University Hospital, Stroke Center, Department of Neurology, Elazığ, Türkiye; ^7^Sakarya University Hospital, Stroke Center, Department of Neurology, Sakarya, Türkiye; ^8^Abant İzzet Baysal University Hospital, Stroke Center, Department of Neurology, Bolu, Türkiye; ^9^Department of Public Health, Amasya University, Amasya, Türkiye

**Keywords:** acute stroke, mechanical thrombectomy, thrombectomy device, first pass complete reperfusion, technique

## Abstract

**Introduction:**

While the Thrombite device differs from the Solitare stent with its Helical open-side structure feature, it shows great similarity with its other features. We assessed the Thrombite device’s effectiveness and safety in this study.

**Materials and methods:**

The study was a retrospective analysis of patients who were included in the Turkish Interventional Neurology database and who had mechanical thrombectomy with the Thrombite device as the first choice between January 2020 and January 2023. The type of study is descriptive research.

**Result:**

Using the Thrombite thrombectomy device, 525 patients received treatment. The median baseline National Institutes of Health Stroke Scale (NIHSS) score was 13, the median initial Alberta Stroke Program Early Computed Tomography (ASPECT) score was 8, and the mean patient age was 68.6+11.7 years. Between the groin puncture and the successful recanalization, the median time was 34 minutes (interquartile range [IQR]: 15–45). 48.2% (modified treatment in cerebral infarction; mTICI) 2b/3% and 33.9% (mTICI 2c/3) were the first-pass recanalization rates. In the end, 87.7% of patients had effective recanalization (thrombolysis in cerebral infarction 2b/3). In the “first-pass” subgroup, the favorable functional result (modified Rankin Scale 0–2) was 51.8%, while it was 41.6% for the entire patient population. The rate of embolization into new territory/different territory were 2.1/0.1%. 23 patients (4.5%) had symptomatic hemorrhage.

**Conclusion:**

The Thrombite device showed a good safety profile and high overall successful recanalization rates in our experience.

## Introduction

The aim of mechanical thrombectomy is to open the vessel quickly and at one time and achieve a successful clinical outcome. Tortuosity of the aortic and/or supra-aortic vessels, the number of passes, clot characteristics and clot load, the techniques and materials used, and the experience of the surgeon affect the rate of vessel opening and clinical outcome (1, 2). An isolated stent retriever, ADAPT, or combined technique can be used as a mechanical thrombectomy technique (3). In randomized clinical studies using third-generation stent retriever devices, both high vessel opening rates (up to 58.7%–88%) and successful clinical outcome rates (32.6%–71.4%) could be achieved (4, 5). In retrospective studies, the rate of opening the vessel with a stent retriever has increased to 97% (6). Also, in studies conducted with 3rd generation thrombectomy devices with different design structures, high vessel opening rates of up to 90–96% have been reported (7–11). The Thrombite device is featured with an S-shaped helical open-side structure, overlapping design, and self-expanding feature, which aims to obtain more efficient clot removal and a higher acute recanalization rate. In the pre-marketing study of the Thrombite device, the acute recanalization rate was reported as 86.5% and 92.3% before and after angioplasty, and the symptomatic intracranial hemorrhage (sICH) within 24 h was as low as 1.9% (12). Our objective in this study is to present the outcomes of patients included in the Turkish Interventional Neurology database who had mechanical thrombectomy with the Thrombite device as the first option.

## Methods

Six stroke sites were identified using prospective data collection from the Interventional Neurology Database between January 2020 and January 2023. We performed a retrospective analysis on 505 consecutive LVO stroke patients. who underwent thrombectomy using a first-line Thrombite device. Patient demographic information, risk factors, procedural details were saved. The type of study is descriptive research.

Each participating site’s institutional review board or ethics committee accepted the study protocol. Prior to enrollment, each patient or their legal representative gave written consent.

### Imaging and patient selection

The study comprised individuals with LVO stroke who were 18 to 96 years old. Noncontrast computed tomography (CT) and contrast-enhanced neck-brain CT angiography were used to confirm LVO. Patients with intracranial hemorrhage were excluded from the study

We established inclusion criteria as follows: pre-stroke modified Rankin Scale (mRS) of 0–1, Alberta Stroke Program Early Computed Tomography (ASPECT) score above 4, angiographically verified LVO in the anterior system, and baseline National Institutes of Health Stroke Scale (NIHSS) score of 4–25. Mechanical thrombectomy was carried out on eligible patients within six hours after the beginning of symptoms (13). Intravenous (IV) tissue-type plasminogen activator (tPA) was given to patients who arrived within 4.5 hours. Patients undergoing CT perfusion were excluded from the study.

### Clinical assessment

Modified thrombolysis in cerebral infarction (mTICI) 2b-3 was used to determine successful recanalization. The mRS was determined at 90 days from discharge. A follow-up CT scan performed within the first 24 hours was used to evaluate the existence of intracerebral bleeding (hemorrhagic infarction type 1, hemorrhagic infarction type 2, parenchymal hematoma type 1, and parenchymal hematoma type 2).

#### Thrombectomy device

Different sizes of the Thrombite™ thrombectomy device are available to accommodate varying anatomical and procedural constraints.

The Thrombite™ “3 mm (3 × 15, 3 × 20, 3 × 25, 3 × 30 mm) and 4 mm (4 × 15, 4 × 20, 4 × 25, 4 × 30” sizes are compatible with microcatheters with a minimum internal diameter of 0.021 inch.

The Thrombite™ “5 mm (5 × 15, 5 × 20, 5 × 25, 5 × 30 mm) and 6 mm (6 × 15, 6 × 20, 6 × 25, 6 × 30” sizes are compatible with microcatheters with a minimum internal diameter of 0.027 inch. In our series, the Headway 21–27 catheter (MicroVention, Valencia, USA) and the excelsior 27 catheter (Stryker Neurovascular, California, USA) were routinely used with all sizes.

The Thrombite™ device is intended for more effective clot removal and optimal revascularization in a variety of vessels. It has an S-shaped helical open-side structure and an overlapped stent that effectively entwine and clamp the clot, assure clot retention, and increase contact surface with thrombus.

A new generation of stent retrievers, the Thrombite has a hybrid closed and open cell architecture that forms a spiral opening along the tubular surface of the device in a longitudinal configuration.

### Thrombectomy technique

Depending on the stroke patient’s health, either local anesthetic or conscious sedation was used throughout the treatment.

For access and flow control in the anterior circulation LVO, either proximal flow arrest or mixed aspiration techniques were applied. Under proximal flow arrest, the Thrombite™ thrombectomy device (Stryker Neurovascular, California, USA) was utilized alone in certain instances. When it was possible to advance the Terumo Destination (Terumo, Shibuya-ku, Japan) into the ICA or the Common carotid artery (CCA), combined aspiration using the Thrombite™ device (Solumbra technique or ADVANCE technique) and a 5 Fr or 6 Fr large-bore catheter was carried out. Depending on the stroke patient’s neurological status, either local anesthetic or conscious sedation was used throughout the treatment.

There were three methods employed: aspiration with large-diameter aspiration catheters, thrombectomy using stent retrievers, or a combination of stent and aspiration approaches.

Using the Thrombite™ thrombectomy device, up to three passes were permitted during the procedure. In the event that effective recanalization was not possible, rescue therapy was permitted. The use of an alternative method (ADAPT), an alternative device (Trevo, EmboTrap, Neva, etc.), balloon angioplasty, or permanent stenting were all considered forms of rescue therapy.

#### Statistical analysis

Following encoding, the data were loaded into the SPSS (Version 22 for Windows, SPSS Inc., Chicago, IL, USA) application for analysis. Data with a normal distribution were represented as mean (standard deviation) for continuous variables, whereas data with an irregular distribution were represented as median (interquartile range) for continuous variables and frequency (%) for categorical variables.

## Results

From January 2020 to January 2023, a total of 575 patients—245 men (48.5%) and 260 women (51.5%)—were treated with Thrombite™, the first-line thrombectomy device. The baseline NIHSS was 14.7 ± 5.1, and the mean age was 68.6 ± 11.7 years. Initial ASPECT score median was 7 (IQR: 5–9). Out of the 505 patients, 105 patients (38.6%) had IV tPA prior to endovascular surgery. We found that 213 patients (42.2%) had M1 occlusion, 127 patients (25.1%) had M2 occlusion, 55 patients (10.9%) had tandem ICA–MCA occlusion, 101 patients (20%) had carotid I, L, and T occlusion, and 9 patients (1.8%) had anterior cerebral artery A1 occlusion. Table 1 summarizes the baseline and demographic features of the patient.

**Table 1:** Baseline characteristics of patients.

Characteristics (*n*: 505)	*n* (%)
Age (mean ± SD)	68.6 ± 11.7
Gender (Male)	245 (48.5)
NIHSS score on admission, median (IQR)	13 (11–18)
CT ASPECT score, median (IQR)	7 (6–9)
**Vascular risk factors, *n* (%)**	
Hypertension	430 (85)
Diabetes mellitus	96 (19)
Atrial fibrillation	162 (32)
Coronary artery disease	56 (11)
Current smoking	126 (25)
Alcoholism	25 (5)
Hyperlipidemia	66 (13)
**Stroke etiology, *n* (%)**	
Cardioembolism	278 (55)
Large artery atherosclerotic disease	49 (9.8)
Carotid dissection	11 (2.2)
Unknown etiology	167 (33)
Intravenous rtPA, *n* (%)	195 (38.6)
**Occlusion site, *n* (%)**	
MCA M1 occlusion	213 (42.2)
MCA M2 occlusion	127 (25.1)
Tandem ICA–MCA occlusion	55 (10.9)
Carotid I, L, T occlusion	101 (20)
Anterior cerebral artery A1 occlusion	9 (1.8)

Values are mean (SD), median (IQR), or *n* (%) as appropriate. ASPECT, Alberta Stroke Program Early CT Score for MCA territory stroke; CT, computed tomography; IQR, interquartile range; NIHSS, National Institutes of Health Stroke Scale.

A groin puncture was performed on average 215 (IQR:155-325) minutes after the last known well or the symptom onset.

A successful recanalization occurred after a groin puncture in 29 (IQR/15-65) minutes on average.

In 48.1% of cases, first-pass recanalization was successful (mTICI 2b/3) and in 33.9% of cases, it was exceptional (mTICI 2c/3). Forty-seven percent of patients (443/505) had successful final recanalization (mTICI 2b/3). The mTICI scores of these patients were 3, 2c, and 2b for 21.6%, 31.5%, and 34.7% of the patients, respectively. Table 2 provides a summary of the reperfusion result.

**Table 2:** Details of endovascular treatment.

Procedural characteristics	
Onset to groin puncture (min)	215 (155–325)
Groin puncture to successful recanalization (min)	29 (15–65)
Balloon guide catheter, *n* (%)	26 (5)
Combined aspiration–stent retriever technique, *n* (%)	479 (94.9)
**Reperfusion outcome, *n* (%)**	
Successful first-pass recanalization (mTICI 2b/3), *n* (%)	243 (48.1)
Excellent first-pass recanalization (mTICI 2c/3), *n* (%)	171 (33.9)
Successful final recanalization (mTICI 2b/3), *n* (%)	443 (87.7)
mTICI 3, *n* (%)	109 (21.6)
mTICI 2c, *n* (%)	159 (31.5)
mTICI 2b, *n* (%)	175 (34.7)
Rescue treatment	39 (7.7)

Values are mean (SD), median (IQR), or *n* (%) as appropriate. IQR, interquartile range; mTICI, modified treatment in cerebral infarction; SD, standard deviation.

In 94.9% of patients, the combined aspiration–stent retriever approach was employed, whereas 5.1% of patients underwent BGC. 39 patients (7.7%) required rescue therapy. In these patients, either trombectomy technique or stentretriever device change was performed.

### Complications

The rate of embolization into new territory was 2.2%. A total of 165 (32.7%) patients had ICH in brain CT at 24th hour. SICH (type 2 parenchymal hematoma) occured in 23 (4.6%) patients. Type 1 parenchymal hematoma was present in thirty-three patients (6.5%), petechial type 1 hemorrhage was present in 39 patients (7.7%), and petechial type 2 hemorrhage was present in 61 patients (12.1%).

A total of 165 (32.7%) patients had ICH in brain CT at the 24th hour. SICH (type 2 parenchymal hematoma) occurred in 23 (4.6%) patients (Table 3).

**Table 3:** Endovascular complications and clinical outcome.

Radiological and clinical outcomes	
Embolization to new territory, *n* (%)	11 (2.2)
**Intracranial hemorrhage, *n* (%)**	
Hemorrhagic infarction 1	39 (7.7)
Hemorrhagic infarction 2	61 (12.1)
Parenchymal hematoma 1	33 (6.5)
Parenchymal hematoma 2	23 (4.6)
Subarachnoid hemorrhage	9 (1.8)
Procedure-related dissection, *n* (%)	8 (1.5)
Vessel perforation	-
**Outcome, *n* (%)**	
Modified Rankin Scale 0–2	262 (41.6)
Modified Rankin Scale 3–6	243 (58.4)
All-cause mortality at 90 days	84 (16.6)

Values are mean (SD), median (IQR), or *n* (%) as appropriate. IQR, interquartile range; SD, standard deviation.

Thirty-three patients (6.5%) had type 1 parenchymal hematoma, 39 patients (7.7%) experienced petechial type 1 hemorrhage, and 61 patients (12.1%) had petechial type 2 hemorrhage.

### Clinical outcome

The percentage of patients who had a good functional outcome (mRS 0–2) was 41.6% in the entire patient population (210/505) and 51.8% (262/505) in the “first-pass” (mTICI 2b/3) sub-group. The overall death rate was 16.6%.

## Discussion

This study presents our retrospective analysis of 505 patients treated in the Turkey Interventional Neurology Database using Thrombite™ as the first-line device. 48.1% of patients had first-pass success (mTICI 2b-3) and 87.7% of patients had successful recanalization (mTICI 2b-3). At 90 days, there was 4.6% symptomatic bleeding and 51.8% (262/505) of the “first-pass” (mTICI 2b/3) sub-group and 41.6% of all patients (210/505) with good functional result (mRS 0–2). Our analysis revealed that thrombectomy with the Thrombite™ device seems safe without major complications.

In both randomized and non-randomized clinical studies published in 2015 and conducted in subsequent years, the superiority of mechanical thrombectomy to medical treatment has been shown. Because with this treatment, both disability and mortality are significantly reduced (13). A recent meta-analysis of randomized clinical trials found that the mortality rate in the treatment group was 16.1%, whereas the control group experienced a mortality rate of 19.2%. At 90 days, the Number-Needed-to-Treat (NNT) required to prevent one death was 32 (14).

With the advent of advanced thrombectomy devices, (the 2nd and 3rd Stent retrievers, modifications in device design) and improvement in thrombectomy technique, an outstanding increase in the rate of successful recanalization has been achieved (15).

In a meta-analysis involving randomized clinical trials, mTICI 2b-3 rates were reported between 58.7% and 88% in thrombectomies performed with the stent retriver technique, while the good clinical outcome at day 90 was between 33-71% (14).

The Thrombite clot retriever device (Ton-bridge, Guangdong, China) was approved by the EU in January of 2020. It has similar features as the 2nd generation stent retriever, such as the overlapping design and self-expanding stent, whereas the helical open-side structure is a unique design (12).

An *in vitro* study showed that the the radial force and flexibility of the Thrombite device were better than the other device (16). In a prospective and randomized study comparing both stents, the clinical outcome, bleeding rates, number of passes, and mortality rates were similar, while at a statistically significant level (*p* < 0.001), the Thrombite device demonstrated a higher rate of successful recanalization (12).

In the same study, the first pass/final mTICI2b/3 rates were 48.1% and 92.3%, respectively, in the group using the Thrombite device. First pass effect was first described by Zaidat et al. (17) and significantly improves clinical outcome. In recent years, the rates of first pass mTICI2b-3 have been increased to 57% with newly-introduced stent retrievers. In the literature, there are different data on the first-pass effect and bleeding complications of different thrombectomy devices (Table 4) (1, 9, 10, 18–22).

**Table 4:** Characteristics of reperfusion therapy studies.

	Patient (*n*)	Device	First pass mTICI 2b-3	Overall mTICI2b-3	sICH
Piasecki et al. (18)	30	Tigertriever	50%	80%	0%
Tomasello et al. (1)	35	ANA	57.1%	91.4%	5.7%
Kaschner et al. (19)	82	Aperio	44.9%	85.3%	7.3%
Lehnen et al. (20)	21	Nimbus	38.1%	76.2%	4.8%
Sakai et al. (21)	11	Versi	45.4%	100%	0%
Srivatsan et al. (9)	70	EmboTrap	35.7%	95.7%	4.3%
Behme et al. (22)	30	ThrombX	80%	93%	0%
Akpinar et al. (10)	118	Neva	56.8%	95,8%	3.3%
Current study	505	Thrombite™	48.2%	87.7%	4.5%

mTICI, modified treatment in cerebral infarction; sICH, symptomatic intracranial hemorrhage.

The Thrombite mechanical thrombectomy device can be used as the first choice for suitable patients.

The main limitation of our study is its non-randomized design and thus lack of a control group. Furthermore, a comparative assessment of retrieval skills per clot type is also not possible because we did not undertake histological analysis of the retrieved clots.

## Conclusion

It is possible to utilize the Thrombite™ thrombectomy device as the first-line option for LVO stroke thrombectomy, given the high rates of first-pass and final recanalization observed in our series. Larger, prospective multi-center trials are necessary to validate our results.

## Data availability statement

The original contributions presented in the study are included in the article/supplementary material, further inquiries can be directed to the corresponding author.

## Ethics statement

Ethical review and approval was not required for the study on human participants in accordance with the local legislation and institutional requirements. Written informed consent from the patients/participants or patients/participants' legal guardian/next of kin was not required to participate in this study in accordance with the national legislation and the institutional requirements.

## Author contributions

ÇA: Writing – original draft. EG: Writing – review & editing. AO: Writing – review & editing. HD: Writing – original draft. AÖ: Writing – review & editing. SY: Writing – review & editing. ZM: Writing – review & editing. EA: Writing – review & editing. BA: Writing – review & editing. MÖ: Writing – review & editing. ÖA: Writing – review & editing. ZU: Writing – review & editing. TA: Writing – review & editing. HE: Writing – review & editing. BT: Writing – review & editing.
